# A gastrin transcript expressed in gastrointestinal cancer cells contains an internal ribosome entry site

**DOI:** 10.1038/sj.bjc.6604326

**Published:** 2008-04-08

**Authors:** A M Grabowska, C A Berry, J Hughes, M Bushell, A E Willis, S A Watson

**Affiliations:** 1 Division of Pre-Clinical Oncology, School of Medical and Surgical Sciences, University of Nottingham, Nottingham, UK; 2 School of Pharmacy, Centre for Biomolecular Sciences, University of Nottingham, Nottingham, UK

**Keywords:** gastrin, translation, gastrointestinal, internal ribosome entry

## Abstract

As the hormone gastrin promotes gastrointestinal (GI) cancer progression by triggering survival pathways, regulation of gastrin expression at the translational level was explored. Sequence within the 5′ untranslated region of a gastrin transcript expressed in GI cancer cells was investigated, then cloned into a bicistronic vector upstream of firefly luciferase and transfected into a series of GI cancer cell lines. Firefly luciferase activity was measured relative to that of a cap-dependent *Renilla* luciferase. A gastrin transcript that was different from that described in Ensembl was expressed in GI cancer cells. Its transcription appears to be initiated within the region designated as the gene's first intron. In GI cancer cells transfected with the bicistronic construct, firefly luciferase activity increased 8–15-fold compared with the control vector, and there was a further induction of the signal (up to 25-fold) following exposure of the cells to genotoxic stress or hypoxia, suggesting that the sequence acts as an internal ribosome entry site. These data suggest that the gastrin transcript within GI cancer cells contains an internal ribosome entry site that may allow continued expression of gastrin peptides when normal translational mechanisms are inactive, such as in hypoxia, thereby promoting cancer cell survival.

Gastrin is normally expressed in G cells of the stomach antrum and regulates both acid secretion and proliferation of gastric mucosal cells (Watson *et al*, 2006). The gastrin gene (*GAST*) is a 4-kb unit consisting of three exons and two introns with the gastrin polypeptide encoded by sequence within exons 2 and 3 (http://www.ensembl.org/index.html). Two different gastrin transcripts have been described in the literature. The transcript given in the Ensembl database was described in human gastrinomas and is a 434-bp transcript incorporating sequence from exon 1 (Ito *et al*, 1984; Wiborg *et al*, 1984). However, another transcript was identified in the gastric antrum that has a transcription start site 111 bp upstream of the start codon (Kato *et al*, 1983). Thus, the 5′ untranslated regions (5′UTRs) of the Ensembl and alternative transcripts are different.

Gastrin upregulation has been shown at both the gene and protein levels in a number of gastrointestinal (GI) (Goetze *et al*, 2000; Mukawa *et al*, 2005; Hur *et al*, 2006) and non-GI cancers (Rehfeld *et al*, 1989; van Solinge *et al*, 1993). At the transcriptional level in GI cancer, gastrin upregulation may be a result of mutational events, for example in the *APC* (adenomatous polyposis coli) or *k-ras* genes (Nakata *et al*, 1998; Koh *et al*, 2000); engagement of the EGF (epidermal growth factor) receptor (Merchant *et al*, 1991); inflammatory events mediated directly by cytokines, such as those associated with *Helicobacter pylori* infection (Suzuki *et al*, 2001; Beales, 2004); or direct activation by certain pathogenicity factors expressed by *Helicobacter pylori* (Rieder *et al*, 2005).

Expression of a number of genes that promote cancer cell survival has been shown to be regulated at the translational level (Pickering and Willis, 2005; Sontheimer and Carthew, 2005). One mechanism, involving the presence of an internal ribosome entry site (IRES) within the 5′UTR of the transcript, may have evolved to allow continued expression of key proteins involved in cell survival during cellular stress (Bushell *et al*, 2004; Holcik and Sonenberg, 2005) when conventional cap-mediated translation is reduced. However, it may also contribute to cancer cell survival as IRESs have been identified in the transcripts of genes that increase proliferation, protect against apoptosis and promote angiogenesis (Vagner *et al*, 1995; Miller *et al*, 1998; Stoneley *et al*, 2000a; Coldwell *et al*, 2001).

Gastrin plays an important role in establishing and supporting the growth of a range of GI tumours (Ferrand and Wang, 2006; Watson *et al*, 2006). As well as acting as a growth hormone, it has well-documented pro-angiogenic (Clarke *et al*, 2006) and anti-apoptotic properties (Konturek *et al*, 2003; Harris *et al*, 2004; Ramamoorthy *et al*, 2004). We have previously used RNAi to downregulate gastrin expression at the gene level and observed a rapid loss of the transcript, but also a delayed downregulation of the endogenous protein compared with GFP-tagged gastrin encoded by a transcript lacking the gastrin 5′UTR (Grabowska *et al*, 2007). This raised the possibility that gastrin expression may be regulated translationally in a manner dependent on the 5′UTR allowing continued protein expression from the small amount of transcript remaining in the cells following RNAi-mediated knockdown. Therefore, we investigated the 5′UTR of the gastrin transcript for sequences that might regulate gastrin expression at the translational level in GI cancer.

## MATERIALS AND METHODS

### Cell culture

PAN1 is a human pancreatic cell line derived from a poorly differentiated human pancreatic adenocarcinoma within the Division of Pre-Clinical Oncology, University of Nottingham (UK). HCT116, a poorly differentiated human colon cell line, was obtained from ECACC (reference no. 91091005). All cell lines were routinely cultured in RPMI 1640 culture medium (Gibco, Paisley, UK) containing 10% (v/v) heat-inactivated fetal bovine serum (FBS; Sigma, Poole, UK) at 37°C in 5% CO_2_ and humidified conditions.

### RNA ligase-mediated rapid amplification of cDNA ends

RNA was extracted from PAN1 cells using RNABee (Biogenesis, Poole, UK) and quantified using an ND-100 Spectrophotometer (Nanodrop, Wilmington, USA). Analysis of the 5′ ends of the gastrin transcripts was carried out using the RLM-RACE kit, according to the manufacturer's instructions (Ambion, Cambs, UK). The sequences of the primers used are given in Table 1. Briefly, 3 *μ*g of total RNA was treated with calf intestine alkaline phosphatase for 1 h at 37°C (to remove free 5′ phosphate groups), phenol-chloroform extracted, isopropanol precipitated, then treated with Tobacco Acid Pyrophosphatase for 1 h at 37°C to remove the 5′ cap. A rapid amplification of cDNA ends (RACE) adapter was ligated to the product using T4 RNA ligase, and cDNA synthesis carried out using random decamers and M-MLV Reverse Transcriptase. A nested polymerase chain reaction (PCR) was carried out using the cDNA as a template. In the first round, the 5′ RACE outer primer was used with the gastrin RACE outer primer. In the second round, the 5′ RACE inner primer was used with the gastrin RACE inner primer.

### TA cloning

The PCR products from the RNA ligase-mediated rapid amplification of cDNA ends (RLM-RACE) nested PCR were cloned into pCRII-TOPO using the TOPO TA cloning kit (Invitrogen, UK) and transformed into TOP10F′ competent cells (Invitrogen). Clones containing inserts were selected by blue-white screening and DNA prepared using Genelute plasmid miniprep kits (Sigma). The DNA was sequenced using a T7 primer and BigDye reaction mix and analysed using an ABI 310 Genetic Analyser (ABI, Warrington, UK). Similarity between the RLM-RACE products and the gastrin gene and transcript sequences obtained from Ensembl Genome Browser (http://www.ensembl.org/index.html) was compared using the ClustalW program in Biology Workbench (http://workbench.sdsc.edu/). The sequence was also submitted to UTRScan (http://www.ba.itb.cnr.it/BIG/UTRScan/).

### Reverse transcriptase-PCR (RT-PCR)

To detect expression of gastrin transcripts, RNA was extracted using the PARIS mirVana kit (Ambion) using the protocol for ‘large’ RNA, then reverse transcribed into cDNA using the Quantitect Reverse Transcription kit (Qiagen, Sussex, UK), which includes a step for removal of genomic contamination. For each RNA, a duplicate reaction was carried out omitting the reverse transcriptase (RT) to ensure that all genomic contamination had been removed. A 2 *μ*l aliquot of the cDNA or ‘RT minus’ control was amplified using a primer specific for the Ensembl transcript (GasEnsF) or the alternative transcript (GasAltF) in combination with a common reverse primer (HGASL). The sequences of the primers are given in Table 1. A 10 *μ*l aliquot of the PCR reaction was analysed on a 2% agarose gel alongside 2-log markers (New England Biolabs, Herts, UK) and visualised using ethidium bromide staining.

To investigate the size of transcripts synthesised from the bicistronic constructs, RNA was prepared using RNABee as described above and cDNA synthesised using Superscript II and random hexamers as described previously (McWilliams *et al*, 1998). To ensure that signals generated were derived from RNA, RT-negative, cDNA reagent and extraction controls were included in which the RT was omitted from the cDNA synthesis, RNA was omitted from the cDNA synthesis or cells were omitted from the RNA extraction. Polymerase chain reaction was carried out using forward and reverse primers located within the *Renilla* and firefly luciferases, respectively, to determine the size of the transcript generated from the plasmid. The sequences of the primers are given in Table 1.

### Reporter construct cloning

The putative gastrin IRES was PCR amplified using forward and reverse primers (*Eco*RI GasIRESF and *Nco*I GasIRESR) designed to contain *Eco*RI and *Nco*I restriction enzyme sites, respectively (shown in lower case in the primer sequences given in Table 1) to facilitate cloning. In the reverse primer, the *Nco*I site was designed to lie over the gastrin start codon, which involved making mutations in the sequence (shown in Table 1 by underlining of altered nucleotides) to create the restriction enzyme site. Following restriction digestion of the PCR product with *Eco*RI and *Nco*I, the fragment was cloned into the bicistronic reporter construct, pRF (Stoneley *et al*, 2000b). This construct contains the *Renilla* luciferase gene downstream of an SV40 promoter and the firefly luciferase gene downstream of a cloning site that contains *Eco*RI and *Nco*I restriction sites. It was also cloned into pBR (Mitchell *et al*, 2005), which lacks a viral promoter. Constructs designated pRGasF and pBRGas were generated. Constructs containing the c-myc IRES (pRMF) and pBRMyc were used as controls (Stoneley *et al*, 2000b).

### Transfections

Transfections were carried out using Lipofectamine 2000 (Invitrogen, Paisley, UK). Test plasmids were co-transfected with a control plasmid (Coldwell *et al*, 2000), encoding *β*-galactosidase (*β*-gal) to provide a transfection control, using a ratio of 5:1, test plasmid: *β*-gal plasmid. Following initial optimisation experiments 500 ng of plasmid was used to transfect 2 × 10^5^ cells in 24-well plates. A 500 ng aliquot of test plasmid plus 100 ng of *β*-gal plasmid were diluted to 50 *μ*l in Optimem (Gibco BRL), mixed with 2 *μ*l Lipofectamine 2000 (diluted to 50 *μ*l in Optimem) for 20 min at room temperature, and added to cells in 500 *μ*l culture medium. To measure basal IRES activity, cells were incubated for 24 h before harvesting. To investigate the activity of the IRES following treatment with mitomycin C (MMC; Sigma) or exposure to hypoxia, medium was changed 6 h after transfection and either normal culture medium, medium containing 2–20 *μ*g ml^−1^ MMC or serum-free medium was added. The cells were then returned to the CO_2_ incubator or exposed to hypoxia (1% oxygen, 5% CO_2_, 37°C) using an Invivo2 400 Hypoxia workstation (Biotrace Fred Baker, Bridgend, UK) for a further 24 h.

Dual transfection of plasmids and siRNAs was carried out by preparing the plasmid transfection mix as above but in half the volume, and preincubating 500 ng of siRNA with 1 *μ*l siPortAmine (Invitrogen) in 25 *μ*l serum-free medium. The two transfection reagents were then mixed immediately before addition to the cells. The *Renilla* siRNA had the target sequence AACGCGGCCTCTTCTTATTTA, which was designed using the Qiagen siRNA Design tool and has a perfect match with *Renilla* luciferase, but nine mismatches and a maximum of six adjacent matching nucleotides when aligned with the sequence of firefly luciferase.

### Reporter assays

Cells were harvested and analysed using the Stop and Glo luciferase assay (Promega). Cells were lysed in PLB buffer (Promega) and both firefly and *Renilla* luciferase activity measured using a MicroLumi XS luminometer (Harta Instruments, Gaithersburg, Maryland, USA). *β*-Gal activity was measured using the Galacto-Light Plus system (ABI). All luciferase readings were expressed relative to the *β*-gal activity in the same sample.

### Statistics

The Student *t*-test was used to compare luciferase activity in cells transfected with IRES constructs *vs* control vectors or exposure to hypoxia *vs* normoxia. The effect of treatment with increasing concentrations of MMC was analysed using one-way ANOVA with Dunnett's multiple comparison test, with *P*<0.05 considered significant.

## RESULTS

### The gastrin transcript identified by RLM-RACE does not contain the 5′UTR of the transcript defined by Ensembl

**Figure 1:**
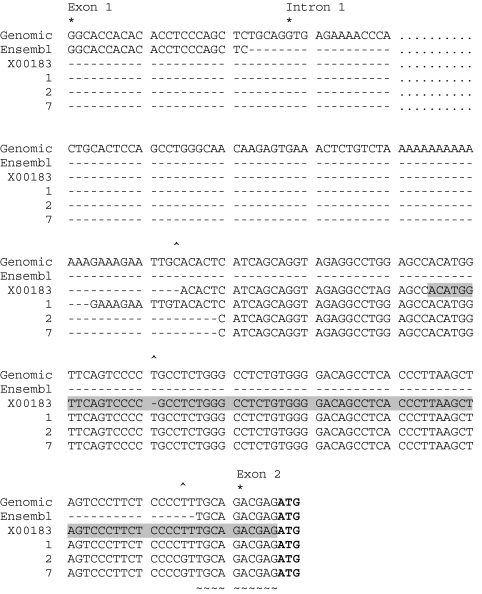
Alignment of Ensembl gastrin genomic sequence (Genomic), Ensembl gastrin transcript (Ensembl), GenBank sequence X00183 (X00183) and three representative RLM-RACE clones (1, 2 and 7). Clones 1 and 7 occurred once each, and clone 2 occurred six times. The beginning of exon 1, intron 1 and exon 2 are indicated by ^*^ and the ATG start codon is highlighted in bold. The central 2860 bp of intron 1 have been omitted for clarity and their position is indicated by - - - - - - - -; only sequence of the first 13-bp and final 171-bp of intron 1 are shown. The RLM-RACE clones have high homology with the region defined by Ensembl as intron 1 and with X00183; ^ indicates positions where one of the clones or X00183 did not match the genomic sequence. There were only 10 nucleotides, which matched the Ensembl transcript, indicated by ∼ below the sequence. The UTRScan-predicted IRES within X00183 is highlighted by shading.

The sequence of the 5′UTR of gastrin transcripts isolated from the pancreatic cell line, PAN1, was analysed by RLM-RACE using two gastrin-specific primers, one in each round of PCR, paired with primers specific for the 5′ adapter sequence. The PCR products were cloned and eight individual clones sequenced. Three unique clones were identified (1, 2 and 7); the remaining clones were identical to clone 2. The sequences of clones 1, 2 and 7 were aligned with the gastrin genomic sequence, the sequence of the 5′UTR of the gastrin transcript provided by Ensembl and the 5′UTR of the transcript described by Kato *et al* (1983) (GenBank entry, X00183). The alignment is shown in Figure 1. There was only a small region of high homology with the 5′UTR of the Ensembl transcript consisting of 10 nucleotides (indicated in Figure 1 by ∼) derived from the end of exon 1 (5 nucleotides) and the beginning of exon 2 (5 nucleotides). However, there was a high degree of homology with the sequence defined by Ensembl as intron 1 and the 5′UTR of the transcript defined in GenBank entry X00183, with differences only at two positions (indicated by ^ in Figure 1); the Ensembl genomic sequence and the RLM-RACE clones all have a T at position −88, which is missing in X00183 and, in addition, the RLM-RACE clones represented by clones 2 and 7 have a G/T substitution at −66. For six of the clones (represented by clone 2 in Figure 1) and in clone 7, the sequence started at a point 106 bp upstream of the ATG codon; for clone 1 the start point was at −122. Thus, the transcript identified by RLM-RACE in the PAN1 cells is different from the gastrin transcript described in Ensembl and more closely resembles that described in GenBank entry, X00183.

### The transcript identified by RLM-RACE is expressed in a range of GI cancer cells

**Figure 2:**
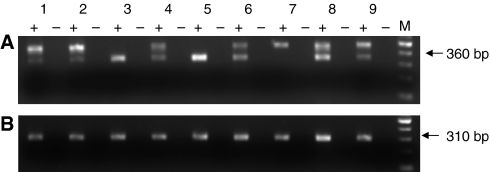
Reverse transcriptase-PCR to investigate expression of the two gastrin transcripts in a panel of GI cancer cell lines: (1) PAN1, (2) BXPC3 (pancreatic); (3) HCT116, (4) HT29 (5) C170HM2 (colonic); (6) ST16, (7) MGLVA1 (gastric); (8) OE19 and (9) OE21 (oesophageal). cDNA (+) and a negative control in which the RT was omitted (−) were amplified using a primer specific for each transcript ((**A**) alternative transcript and (**B**) Ensembl transcript) and a common primer. The expected position of the bands is indicated by an arrow based on the position of the 100-bp markers (M).

RT-PCR was carried out in a panel of GI cancer cells using primers specific for the 5′UTR of the Ensembl transcript or the alternative transcript in combination with a common primer (Figure 2). Both transcripts were present in all the cell lines tested, which included cells of pancreatic, colonic, gastric and oesophageal origin. For the alternative transcript, two amplification products were observed: a band of the predicted size of 360 bp in all of the cell lines except for MGLVA1 cells and a higher molecular weight product of 490 bp in all of the cell lines except HCT116 and C170HM2 cells. The size of this larger product equates to the expected transcript but with the intron 2 retained. However, this does not appear to be derived from amplification of contaminating genomic DNA as it did not appear in the RT-negative control.

### Prediction of an IRES within the gastrin transcript identified by RLM-RACE

The 5′UTR of the transcript identified in the GI cancer cells by RLM-RACE is GC-rich, with a 60.7% GC content and includes an AUG upstream of the translation start site. Therefore, we analysed the sequence for elements that might regulate the stability of the transcript or its translation. The sequences of the 5′UTRs of the gastrin transcript identified by RLM-RACE, the Ensembl transcript and the transcript described in X00183 were submitted to UTRScan. An IRES, consisting of 91 bp of sequence upstream of the start codon, was predicted to be present only in the X00183 gastrin transcript (sequence shaded in Figure 1), in spite of the homology between the X00183 transcript and that identified by RLM-RACE, the RLM-RACE transcript differing only by the inclusion of one additional T at −66.

### Assessment of the activity of the putative IRES to regulate translational activity

To assess the ability of the putative IRES to regulate translation a bicistronic reporter vector, pRF was used, consisting of two luciferase genes encoded within a single transcript expressed under the control of an SV40 promoter. The predicted IRES was amplified and cloned into pRF to generate a construct pRGasF. The sequence of two clones was compared with the sequence of the transcript predicted in X00183. The additional T nucleotide at position −66 was found to be present in both clones, as expected from the RLM-RACE data; in addition, some minor differences were found at positions −1/2 and −96/97.

**Figure 3:**
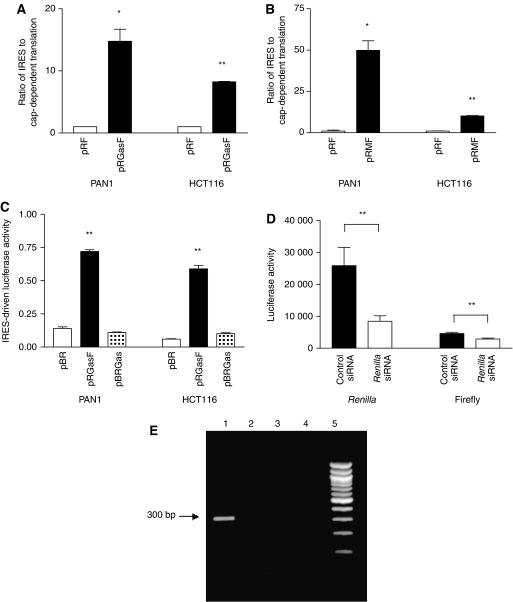
Activity of the putative gastrin IRES and Myc IRES in pancreatic (PAN1) and colon (HCT116) cancer cell lines. Basal activity of (**A**) the gastrin IRES (pRGasF) or (**B**) Myc IRES (pRMF) compared with empty vector (pRF). (**C**) Activity of the gastrin IRES in a promotorless plasmid (pBRGas) compared with the empty vector (pBR) or pRGasF. (**D**) Significant knockdown of both *Renilla* and firefly luciferase activity in HCT116 cells transfected with pRGasF and a *Renilla* siRNA, compared with cells transfected with pRGasF and a control siRNA (*P*<0.005 for both). (**E**) Reverse transcriptase-PCR of cells transfected with pRGasF using forward and reverse primers located within the *Renilla* and firefly luciferase sequences. A single band of ∼300 bp was observed (Lane 1). No bands were observed in the RT-negative, cDNA reagent and RNA isolation controls (Lanes 2–4). Lane 5: markers. Statistical significance is indicated by ^*^P<0.05 or ^**^P<0.01.

The 5′ *Renilla* luciferase in the bicistronic vector was designed so it would be translated in a cap-dependent manner but translation of the 3′ firefly luciferase would be dependent on the insertion of an IRES. For each construct, the activity of the IRES-dependent luciferase was measured relative to the empty vector and cap-dependent luciferase signal in Pan1 and HCT116 cells. There were significant 8- and 15-fold increases (*P*=0.02 and *P*<0.001) in luciferase activity in PAN1 and HCT116 cells, respectively, following insertion of the putative gastrin IRES (pRGasF). A construct containing the c-myc IRES (pRMF) was included as a positive control in these experiments (Figure 3A and B). The activity of this IRES was particularly high in PAN1 cells with a 49-fold induction of activity (*P*=0.014) but, in HCT116 cells, the activity was similar to that of the gastrin IRES, with a 10-fold increase compared with the empty vector, pRF (*P*=0.007).

### The putative gastrin IRES has no activity in a plasmid lacking a promoter

To verify that the putative gastrin IRES is not acting as a promoter in pRGasF, it was cloned into a reporter vector, pBR, lacking a viral promoter but with viral enhancer elements still present downstream of the firefly luciferase. There was no increase in luciferase activity in the pBR construct when the putative gastrin IRES was inserted (pBRGas), suggesting that the sequence is not able to act as a promoter, even in the presence of viral enhancer sequences while activity of the pRGasF IRES construct was as expected (Figure 3C). In addition, when a *Renilla* siRNA was used to treat the pRGasF-transfected cells, there was a significant knockdown of the firefly luciferase activity in parallel with the knockdown of the *Renilla* activity (*P*<0.005 for both), suggesting that both are encoded on the same transcript (Figure 3D), and RT-PCR of RNA from pRGasF-transfected cells using primers located within the *Renilla* and firefly luciferases, respectively, revealed a single band of approximately 300 bp (Figure 3E), which equates to a full-length RNA without smaller splice variants.

### The activity of the putative gastrin IRES is upregulated following exposure of the cells to genotoxic stress or hypoxia

**Figure 4:**
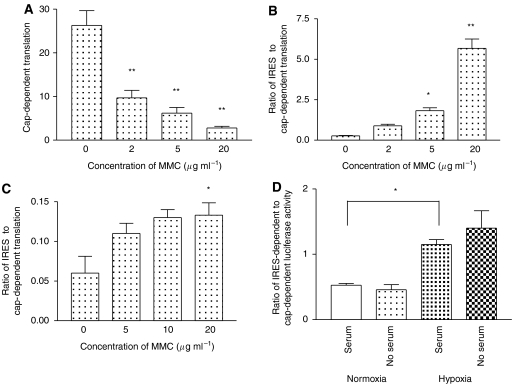
Effect of MMC and hypoxia on IRES activity. Treatment with MMC significantly reduced cap-dependent translation of the *Renilla* luciferase (**A**) but resulted in an increase in the ratio of IRES-dependent to cap-dependent translation in both HCT116 (**B**) and PAN1 cells (**C**). Similarly, 24 h exposure to a hypoxic environment increased IRES-dependent translation (**D**). Statistical significance is indicated by ^*^P<0.05 or ^**^P<0.01.

Since a role for gastrin in promoting cell survival has been shown, the activity of the IRES was studied after exposure of HCT116 and PAN1 cells transfected with pRGasF to a range of concentrations of the DNA-damaging agent, MMC. As expected, with increasing concentrations of MMC, activity of the cap-dependent luciferase was switched off (Figure 4A). However, activity of the IRES-driven luciferase was either maintained or increased resulting in an increase in the ratio of firefly to *Renilla* luciferase activity in both cell lines (Figure 4B and C). This suggests that the putative gastrin IRES is able to support translation under circumstances where cap-dependent translation is not functional.

Gastrin is also known to promote angiogenesis, and so we investigated the effect of hypoxia on IRES-driven luciferase activity. The effect of serum was incorporated into this study as the activity of the HIF IRES has previously been shown to be most active in serum-free conditions. In HCT116 cells, there was a significant increase in the relative activity of the IRES-driven compared with the cap-driven luciferase after 24 h exposure to hypoxia in the presence of serum (*P*=0.016). There was also an increase in the absence of serum, but this did not reach statistical significance (*P*=0.078).

## DISCUSSION

We have shown that a gastrin transcript expressed in a panel of GI cancer cell lines contains an IRES that has basal activity in both pancreatic and colon cancer cells. The sequence appears to be acting as a true IRES, as activity is lost in the absence of a viral promoter. Under conditions of cellular stress, activity of the IRES is increased or maintained at higher levels than cap-dependent translation suggesting that it is controlled by a distinct mechanism, as shown for the c-myc IRES (Subkhankulova *et al*, 2001).

The IRES-containing transcript found in the GI cancer cell lines appears to use a transcription start site within the sequence assigned by Ensembl as intron 1 and is homologous to the transcript described in G cells (Kato *et al*, 1983). These authors localised a possible transcription start site by S1 nuclease mapping, following hybridisation with gastric antrum polyA RNA, to approximately 111 bp upstream of the start codon. Potential Goldberg-Hogness and CCAAT boxes were found upstream of the transcription start site in the 5′ flanking DNA sequence (Kato *et al*, 1983).

Seven of the independent clones generated by RLM-RACE started 106 bp upstream of the ATG start codon and the remaining clone started a further 16 bp upstream. It is unlikely that the products arise from amplification of DNA fragments; such fragments arise at random and are unlikely to have 5′ ends, which cluster at a single location. In addition, RLM-RACE is designed to prevent amplification of DNA templates; it includes steps which remove free 5′ phosphates from uncapped RNAs and contaminating DNA, preventing ligation of the adapter to such nucleic acids, and no product was detected when the enzyme TAP, which removes the cap to reveal the free 5′ phosphate on mRNA and allow ligation of the adapter, was omitted.

In addition, using RT-PCR, the alternative transcript was shown to be present in all of the GI cancer cell lines investigated including cells of pancreatic, colonic, gastric and oesophageal origin. An amplification product with a higher molecular weight than that predicted was also amplified in most of the GI cancer cell panel using a primer specific for the alternative transcript; this appears to have retained intron 2 and further investigation will be required to determine how this transcript arises and whether it is functional. However, the predicted transcript was also present in the majority of the cell lines investigated; while the intensity of the band derived from the Ensembl transcript was fairly uniform across the cell line panel, the intensity of the bands from the alternative transcript varied between cell lines.

This raises the question of how transcription of these two mRNAs is regulated. Most studies investigating regulation of gastrin gene transcription in GI cancer cells have used constructs based on the promoter associated with the Ensembl transcript. Further studies to investigate the DNA flanking sequence will be required to identify the promoter, that drives transcription of this alternative mRNA. Transcription from alternative start sites has been shown previously in certain genes, including oncogenes, giving rise to transcripts that are differentially regulated at the translational level; for example, *c-myc* has four alternative transcripts driven by different promoters and only one of these contains an IRES (Marcu *et al*, 1992).

The 5′UTR of the alternative gastrin transcript described in the present study is GC-rich, in keeping with other described IRESs, which are generally highly structured (Pickering and Willis, 2005). Since the function of IRESs is probably dependent on their three-dimensional structure rather than the presence of specific sequences, it has been difficult to identify motifs that allow their definitive identification. A recent paper has identified a motif, (CCU)_*n*_ occurring within a polypyrimidine tract, which acts as an artificial IRES when the polypyrimidine tract protein, one of the *trans*-acting factors thought to be involved in initiation of translation from IRESs, is present (Mitchell *et al*, 2005). We used a bioinformatics-based method to look for common functional sequences that occur in the UTRs of transcripts (Pesole and Liuni, 1999), which includes a search for a motif common to IRESs of cellular RNAs that forms a Y-type stem loop structure (Le and Maizel, 1997). Interestingly, while UTRScan identified a potential 94-bp IRES within the 5′UTR of the transcript described in GenBank entry X00183, it did not identify an IRES within the 5′UTR of the transcript identified by RLM-RACE, which differs only by insertion of a single nucleotide 66 bp upstream of the start codon, showing that such programs can only be used to provide guidance.

Therefore, we tested the ability of the predicted IRES to drive translation within a bicistronic reporter construct (Stoneley *et al*, 1998; Coldwell *et al*, 2001; Mitchell *et al*, 2001). There was significant IRES-dependent activity in both of the GI cancer cell lines used. In PAN1 cells, the IRES-driven luciferase activity was lower than that observed using a construct containing the c-myc IRES. In HCT116 cells, while the activity of the gastrin IRES was lower than in PAN1 cells, it was higher than that of the c-myc IRES in these cells. For these experiments, we inserted only the 94-bp sequence, which was predicted to be an IRES by UTRScan and used the sequence variant found in the PAN1 gastrin transcript. It is possible that the removal of the inserted T and use of the whole of the transcript's 5′UTR might lead to a higher level of activity.

The luciferase activity observed when the gastrin 5′UTR sequence was inserted into the bicistronic construct might occur because the sequence is able to act as a promoter and allow independent transcription of the downstream luciferase. However, when the gastrin sequence was cloned upstream of luciferase in a vector that did not contain a promoter, there was no luciferase activity compared with the empty vector. This is further suggested by the reduction in firefly luciferase activity observed in parallel with the reduction in *Renilla* luciferase activity following treatment with a *Renilla* luciferase-specific siRNA. Another possible explanation for the activity of the luciferase downstream of the putative gastrin IRES is that the sequence acts as a splice acceptor, as has been suggested for the XIAP sequence (Van Eden *et al*, 2004), but we did not observe any smaller RT-PCR products to indicate that the IRES sequence was being removed by splicing. Taken together, these data suggest that the gastrin sequence is acting as an IRES.

Under conditions of cellular stress, for example apoptosis, cap-dependent translation is switched off as a result of cleavage of initiation factors such as EIF4G and EIF4B, which prevents circularisation of the transcript (Spriggs *et al*, 2005). Expression of genes that play an anti-apoptotic role in cancer has been shown to be maintained via use of an IRES (Coldwell *et al*, 2001), and since such a role has also been ascribed to gastrin in GI cancer cells (Harris *et al*, 2004), we investigated whether the gastrin IRES was active during apoptosis induced by the genotoxic agent, MMC. Gastrin also has a role in promoting angiogenesis and so, in parallel, we investigated the effect of hypoxia on the gastrin IRES activity. In both cases, activity of the IRES was increased relative to cap-dependent translation.

The translation initiation factors that regulate expression from IRESs have begun to be elucidated. They appear to include canonical translation initiation factors such as eIF2, eIF3 and eIF4F (Pestova *et al*, 1996) and also other *trans*-acting factors, and may act to change the secondary structure of the IRES to facilitate ribosomal binding (Mitchell *et al*, 2003; Pickering *et al*, 2004). Differential expression of such factors may explain the different patterns of activity of individual IRESs during development and differentiation (Sella *et al*, 1999; Creancier *et al*, 2000; Mitchell *et al*, 2003) and in cancer (Evans *et al*, 2003). In the present study, the basal activity of the *c-myc* and gastrin IRESs in PAN1 and HCT116 cells, which are of pancreatic and colonic origin, did not correlate. In addition, the activity of the *c-myc* IRES, which has previously been shown to be upregulated by MMC in HeLa cells (Subkhankulova *et al*, 2001), was not increased following exposure of these GI cancer cells to MMC, but it was responsive to hypoxia (data not shown). Taken together, these data suggest that expression from the two IRESs is controlled independently, involving different *trans*-acting factors.

Expression of a transcript containing an IRES, which allows continued expression of gastrin peptides in the face of apoptotic stimuli or hypoxia would promote the survival of GI cancer cells. Thus, further investigation of the mechanisms regulating expression of the transcript and activity of the IRES in different cell types is warranted.

## Figures and Tables

**Table 1:** Primer sequences

**Primer**	**Sequence**
RACE adapter	GCUGAUGGCGAUGAAUGAACACUGCGUUUGCUGGCUUUGAUGAAA
5′ RACE outer primer	GCTGATGGCGATGAATGAACACTG
Gastrin RACE outer primer	TCCAGCCAGGGTAGCTCCAG
5′ RACE inner primer	CGCGGATCCGAACACTGCGTTTGCTGGCTTTGATG
Gastrin RACE inner primer	GGCTTCCAAGAAGCTTCAGA
GasEnsF	CCACACACCTCCCAGCTC
GasAltF	TGGAGCCACATGGTTCAGT
HGASL	TCCATCCATCCATAGGCTTC
*Eco*RI GasIRESF	CCGCCGgaattcAGGCCTAGAGCCACATGGT
*Nco*I GasIRESR	ACACACACAGTCGCTccatggCGTCTGCAA
*Renilla* luciferase F	GCAAGAAGATGCACCTGATG
Firefly luciferase R	GCGTATCTCTTCATAGCCTT
